# Editorial: Proceedings of ICPSBBB 2018 - 2^nd^ International Conference on Plant Synthetic Biology, Bioengineering and Biotechnology

**DOI:** 10.3389/fpls.2020.614933

**Published:** 2020-11-02

**Authors:** Henrik Vibe Scheller, Nicola Patron, Poul Erik Jensen

**Affiliations:** ^1^Environmental Genomics and Systems Biology Division, Lawrence Berkeley National Laboratory, Joint BioEnergy Institute, Berkeley, CA, United States; ^2^Department of Plant and Microbial Biology, University of California, Berkeley, Berkeley, CA, United States; ^3^Engineering Biology, Earlham Institute, Norwich Research Park, Norfolk, United Kingdom; ^4^Department of Food Sciences, University of Copenhagen, Frederiksberg, Denmark

**Keywords:** synthetic biology, programmable genetic circuits, plant genomes, transgene expression, transformation efficiency, CRISPR/Cas9, bioproduction, scaffold proteins

Plant synthetic biology combines engineering principles with plant biology toward the design, construction, and testing of plants with new traits (Liu and Stewart, 2015). Synthetic biology applies engineering principles, using defined genetic parts with the aim of achieving predictable outcomes. The field aims at providing fuels, pharmaceuticals, bioactive metabolites and increased crop yield. Synthetic biology is increasingly forming a framework in genetic transformation of plants allowing us to improve traits and crop performance. The conference on *Plant Synthetic Biology, Bioengineering, and Biotechnology* covers broad aspects of plant synthetic biology, ranging from tools to the design of predictable functions and precision genome editing, to re-designed photosynthesis, plant bioengineering techniques, and rational design of novel plant traits.

This Frontiers in Plant Science virtual issue on the “Research Topic Proceedings of ICPSBBB 2018 - 2nd International Conference on Plant Synthetic Biology, Bioengineering and Biotechnology” consists of 8 publications, including four reviews and four original research articles, which fall into the following general topics: quantitative and genetic parts, evolution, expression of transgenes and epigenetic changes, engineering of stress tolerance and bioproduction.

## Quantitative and Genetic Parts

A primary goal of plant synthetic biology is to produce predictable and programmable genetic circuits from simple regulatory elements and well-characterized genetic components. McCarthy and Medford provide a detailed review of the development of quantitative and predictive genetic parts for plant synthetic biology. In this context, the quantitative properties and transfer function of a variety of genetic parts are needed which can be aided by computer based selection of the optimal components to be assembled into functional devices. The review highlights challenges with synthetic biology approaches in complex organisms like plants composed of differentiated cells and tissues, with unique regulatory or developmental contexts affecting the introduced synthetic genetic circuits. Being able to measure the function of plant components within the context of a living plant cell will be essential for exploiting these components in gene circuits with predictable function. The need for mathematical modeling to account for variable contexts is discussed.

In the research paper by Persad et al., the need to develop technologies to precisely regulate gene expression and engineer complex genetic circuits into plant chassis is addressed experimentally. The Q-system from *Neurospora crassa*, which is a powerful repressible- and engineerable-binary system, has been repurposed in a variety of eukaryotic systems. The functionality of the Q-system in plants is demonstrated using transient expression in soybean protoplasts and in *Nicotiana benthamiana* leaves. The modulated expression of reporter genes is demonstrated as is a potential application for plant-based biosensors, thus demonstrating that the Q-system has potential as a powerful orthogonal tool for precise control of gene expression in plants.

## Evolution, Expression of Transgenes, and Epigenetic Changes After Gene Editing

Plant genomes are complex and the number of transcription factors is large and functionally divergent. How the evolutionary network rewiring process, regulatory gene duplication followed by functional divergence, can be used to inspire synthetic biology approaches in order to develop novel phenotypic variation to be used in future trait based breeding programs in plants is reviewed by Law et al..

During generation of transgenic plants the genomic insertion site has widely been thought to be crucial for success. Betts et al., evaluate the importance of transgene insertion site in maize and soybean using both random and site-specific transgene integration. The relative contribution of genomic location on transgene expression levels with other factors, including cis-regulatory elements, neighboring transgenes, genetic background, and zygosity is compared. It is demonstrated that cis-regulatory elements and the presence/absence of nearby transgene neighbors can impact transgene expression. Surprisingly, however, the genomic location had the least impact on transgene expression compared to the other factors that were investigated. The majority of insertion sites recovered supported transgene expression levels that were statistically not distinguishable.

Transformation of cereals is still challenging, and in barley, *Agrobacterium*-mediated transformation efficiency is highly dependent on genotype with very few cultivars being amenable to transformation. Orman-Ligeza et al., found that cultivars with large embryos were more amenable to transformation, and the *TRA1* locus, was found to be responsible for transformation efficiency. The hunt is on for identification of *TRA1*, which is so far mapped to a region containing 225 genes on chromosome 2H.

CRISPR/Cas9 has revolutionized the pace of plant genome editing and precision breeding in crops. Lee et al., investigated the degree and patterns of epigenetic changes after gene editing. DNA methylation in genome-edited promoters of naturally hypermethylated and hypomethylated genes from *Arabidopsis* was studied using bisulfite sequencing comparing paired groups of edited and non-edited plants to identify changes in DNA methylation of the targeted loci. It was found that directed mutagenesis via CRISPR/Cas9 resulted in no unintended morphological or epigenetic alterations. Off-target mutations were also not detected.

## Engineering of Stress Tolerance and Bioproduction

Engineering stress tolerance in plants has huge potential to accelerate and keep pace with the effects of climate change. In the review by Lohani et al., the current knowledge of stress-responsive genes and their role in imparting multiple stress tolerance in *Brassica napus* (rapeseed/canola) is presented in the context of network cross-talk through omics data mining to unravel the underlying complexity required for stress sensing and signaling. Transgene-free genome editing and utilization of nanoparticles as gene delivery tools are discussed as tools to introduce the relevant traits.

Peng et al., summarize recent efforts in obtaining plant-derived biosynthetic and the extracellular matrix protein, collagen. These products have potential use as scaffold proteins for biomatrices. Spider silk analogs and collagen are composed of a large number of tandem block repeats, which together with the need for post-translational modifications makes production in bacterial hosts challenging leaving plants as an alternative production host.

## Author Contributions

HVS, NP, and PEJ co-edited the Research Topic. All authors wrote, edited, and approved the final version of the Editorial.

## Conflict of Interest

The authors declare that the research was conducted in the absence of any commercial or financial relationships that could be construed as a potential conflict of interest.

## References

[B1] Liu W. Stewart C. N Jr. (2015). Plant synthetic biology. Trends Plant Sci. 20, 309–317. 10.1016/j.tplants.2015.02.004 25825364

